# Prevalence of dermatological, oral and neurological problems due to face mask use during COVID-19 and its associated factors among the health care workers of Bangladesh

**DOI:** 10.1371/journal.pone.0266790

**Published:** 2022-04-12

**Authors:** Sreshtha Chowdhury, Simanta Roy, Mohammad Azmain Iktidar, Shahidur Rahman, Mowshomi Mannan Liza, A. M. Khairul Islam, Sharmin Akhter, Madhuritu Bhadra Medha, Afia Tasnim, Antara Das Gupta, Auditia Deb, Shresta Chowdhury, Mohammad Delwer Hossain Hawlader

**Affiliations:** 1 Department of Public Health, North South University, Dhaka, Bangladesh; 2 Public Health Professional Development Society (PPDS), Dhaka, Bangladesh; 3 Chittagong Medical College and Hospital, Chattogram, Bangladesh; 4 ZH Sikder Women’s Medical College & Hospital, Dhaka, Bangladesh; 5 Rangamati Medical College, Rangamati, Chattogram, Bangladesh; 6 Dhaka Medical College, Dhaka, Bangladesh; Indian Institute of Technology Bombay, INDIA

## Abstract

**Background:**

When caring for COVID-19 patients, using personal protective equipment (PPE) may significantly lower the risk of infection of health care workers (HCWs). However, adverse responses due to PPE use have been observed during the 2003 SARS pandemic. This study will highlight the different adverse reactions caused by face mask use, one of the essential components of PPE in the HCWs, and identify the factors associated with these problems.

**Methods:**

This cross-sectional survey was conducted between September and October 2021. 404 HCWs were selected by snowball sampling from four randomly selected healthcare facilities of Bangladesh. Trained volunteers collected data by face-to-face interview using a pretested structured questionnaire. Data were analyzed using STATA (v.16) and summarized using frequency and relative frequency. Later, the chi-square test was used to explore bivariate relationships, and the binary logistic regression model was fit to identify the predictors.

**Results:**

The majority of the respondents were 26–36 years (70.30%), male (69.80%), and doctors (74.50%). 48.76% of the respondents had unfavorable skin responses beneath the face masks; female gender, physicians, professionals working more than 32 hours a week, wearing N95, and more than one mask were predictors of skin problem. 28.47% and 60.15% of all participants suffered from some form of oral and neurological problems, respectively.

**Conclusion:**

Face mask use sequelae, especially skin, oral and neurological problems, are prevalent among health care workers. Therefore, necessary precautionary measures should be taken to safeguard our frontlines.

## Introduction

Covering mouth and nose as a part of conventional hygiene practices can be dated back to early modern Europe. The main goal of this precaution was to neutralize the so-called miasma in the air by wearing scents and spices under a mask, similar to the plague doctors’ bird-like masks. However, these practices lost steam by the eighteenth century, and face mask use as we know it today can be traced back to a more recent past [1]. Following Carl Friedrich Flügge’s works on the development of droplet infections in 1897 [2], Flügge and Johannes von Mikulicz wrote a paper describing the usage of protective face masks in operating rooms. One layer of gauze was used as a "mouth bandage," which was meant to shield the patient from wound infection [3]. Face masks are now worn not only during the surgery but also to prevent the spread of respiratory viruses from one person to another [4] and were crucial during Swine flu (2009) and SARS (2003) pandemics [5].

A new coronavirus (SARS-CoV-2) causing severe acute respiratory syndrome symptoms was declared a public health crisis by WHO on January 30, 2020. The disease was later named coronavirus disease 2019 (COVID-19) and announced a pandemic on March 11, 2020 [6, 7]. According to epidemiologic data, droplets produced during face-to-face encounters when talking, coughing, or sneezing appear to be the most common transmission mechanism [8]. Since health care workers (HCWs) come in direct contact with COVID-19 patients, they are prone to danger from this highly infectious virus [9]. When caring for COVID-19 patients, using personal protective equipment (PPE) may significantly lower the risk of infection [10]. Although PPE use prevents disease spread, it also resulted in certain symptoms and adverse responses due to the prolonged use, as was previously observed during the 2003 SARS pandemic [11].

Acne, rash, itching, xerosis, and nasal bridge scarring have been documented in face mask users [11–13]. The most frequently reported adverse skin responses (68.9%) among HCWs who used N95 masks were nasal bridge scarring and face itching (27.9%) [12]. Global healthcare providers also faced a scarcity of face masks during the COVID-19 pandemic, which led to their extensive re-use and, as a consequence, various adverse reactions [14]. Many more HCW might have had skin responses but were self-medicated and did not seek medical attention unless the reaction was very severe [11]. N95 mask users are also reported to be suffering from headaches and attention deficit [6]. Given the fact that several aspects of face mask related health consequences remain unknown, this study evaluated the effect of several face mask-related symptoms among HCW. This study also investigated possible predictors of those problems.

## Materials and methods

### Study design and study site

This cross-sectional survey was conducted in Bangladesh between September and October 2021. One government hospital, one private hospital, and one dental clinic were randomly selected from a list of hospitals and dental clinics from each of the eight divisions (Dhaka, Chittagong, Rajshahi, Barisal, Mymensingh, Rangpur, Khulna, and Sylhet). A military healthcare facility was also selected similarly from a list. This selection procedure was reconsidered if any of the selected facilities did not consent verbally. This article was prepared in accordance with the checklist of the Strengthening the Reporting of Observational Studies in Epidemiology (STROBE statement) [15].

### Study participants

**Fig 1:**
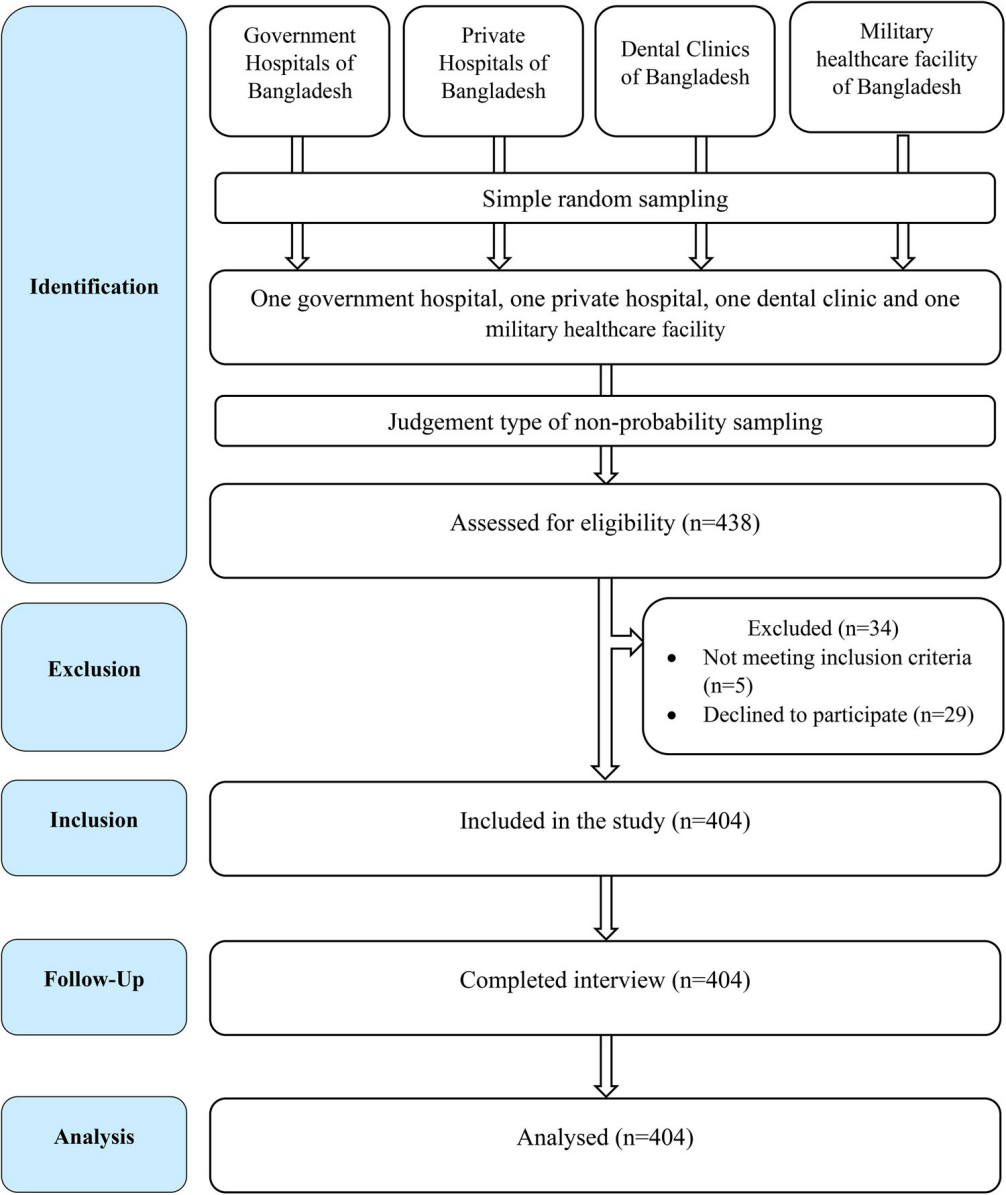
STrengthening the Reporting of OBservational studies in Epidemiology (STROBE) flow chart of study participants.

A total of 438 healthcare workers were approached from these institutes using the judgement type of non-probability sampling technique. Registered doctors, nurses, technologists, and assistants who used any face mask were included in the study. Participants with pre-existing history of skin, oral and neurological problems prior to facemasks use during COVID-19 pandemic, refusing to participate in this study and foreign nationals were excluded, considering genetic and cultural diversity. Five healthcare workers were excluded due to not meeting the inclusion criteria and 29 were excluded for not providing consent, leaving a total sample size of 404 healthcare workers (Fig 1).

### Data collection

Volunteers were selected from each of the randomly selected study sites and trained accordingly. Volunteers conducted face-to-face interviews maintaining recommended social distancing protocol with the participants after obtaining verbal consent. Written consent were not taken to avoid physical contact as the participants were working in places with close contact to COVID-19 patients. A telephone interview was considered for those who were duty in COVID-19 dedicated unit or home-quarantined.

### Study instrument

An initial questionnaire was prepared by the researchers based on the review of the previous research. It was then pretested on 30 participants, and based on their recommendation final version was adopted. The questionnaire was translated to Bengali from English using ISPOR (International Society for Pharmacoeconomics and Outcomes Research) translation guideline [16]. Respondents were asked about any new problems due to wearing a mask during the COVID-19 pandemic. From the response, the common and relevant issues were included by the researchers. Respondents were also asked about their socio-demographic situation (age, sex, occupation, workplace), duration of mask use (≤12 months, >12 months), average mask use per week (<32 hours, 32–56 hours, >56 hours). Moreover, type of face mask they had used (N95, KN95, surgical, cloth mask, other masks), simultaneous multiple mask use, history of COVID-19 infection and vaccinations, availability of workplace cooling system were also included in questionnaire.

### Statistical analysis

We used Stata (version 16; StataCorp, College Station, TX, USA) for data analysis. A histogram, a normal Q-Q plot, and the Kolmogorov-Smirnov test were used to check for normality in continuous data. Arithmetic mean was used for quantitative data as a measure of center and standard deviation was used as measure of dispersion. Categorical variables were expressed as frequency with relative frequency. Pearson’s chi-square test was used to explore the bivariate relationship between categorical predictor and outcome variables. The outcome variables (dermatological problem, oral problem & neurological problem) was coded as 0 = ‘No problem’ and 1 = ‘Problem’ and three separate binary logistic regression models were fit to determine the factors that are linked to each of the outcomes, after adjusting for confounders. We further estimated variance inflation factor to check for multicollinearity. A p-value of <0.05 was considered statistically significant.

### Ethical clearance

This study was approved by the Institutional Review Board (IRB) of North South University (Approval no 2021/OR-NSU/IRB/1001) and adhered to the 1975 Declaration of Helsinki’s ethical criteria (6th version, 2008), as shown in a priori approval by the institutional review committee. Verbal informed consent was obtained from all the participants to avoid physical contact as the participants were working in places with close contact to COVID-19 patients and it was approved by the IRB of North South University. Once the participants consented to voluntary participations, the volunteers took a note. Our study didn’t include any minors.

## Results

### Participant characteristics

A total of 404 participants responded in this study, and most of them (65.84%) spent over nine months in humid areas. The majority (70.30%) of the respondents were 26–36 years and male (69.80%). In this study, doctors’ were the majority (74.50%), followed by the nurse (12.62%) and technologists (7.67%). 42.33% of professionals worked in government healthcare facilities, and the rest worked in private facilities, dental chambers, ICU, and other institutions. Nearly two-thirds of the participants did not have air conditioning in their workplace. Around four-fifths of the respondents have used face masks for over 12 months and three-fifths used face masks ≥32 hours a week on average. There were four types of masks most commonly used in the participants, surgical masks (347; 85.89%), N95 masks (196; 48.51%), KN95 masks (148; 36.63%), and cloth masks (55; 13.61%). 73% of the participants used multiple masks occasionally, and 55% did not use the same mask twice. Less than half of the participants had a previous history of COVID-19 infection, and 80% were fully vaccinated (Table 1).

**Table 1:** Baseline characteristics of study participants.

Variable	N (%)
**Age** ^a^	
<26 years	80(19.80)
26–36 years	284(70.30)
> 36 years	40(9.90)
**Occupation** ^a^	
Doctor	301(74.50)
Nurse	51(12.62)
Technologist	31(7.67)
Others	21(5.20)
**Sex** ^a^	
Female	122(30.20)
Male	282(69.80)
**Workplace** ^a^	
Unemployed	35(8.66)
Government facilities	171(42.33)
Private facilities	104(25.74)
Dental chamber	32(7.92)
ICU	3(0.74)
Others	59(14.60)
**Duration of mask use** ^a^	
≤12 months	89(22.03)
>12 months	315(77.97)
< 32 hours	163(40.35)
32–56 hours	143(35.40)
> 56 hours	98(24.26)
**Facemask type**	
**N95** ^a^	
No	208(51.49)
Yes	196(48.51)
**KN95** ^a^	
No	256(63.37)
Yes	148(36.63)
**Surgical** ^a^	
No	57(14.11)
Yes	347(85.89)
**Cloth mask** ^a^	
No	349(86.39)
Yes	55(13.61)
**Other mask** ^a^	
No	379(93.81)
Yes	25(6.19)
**Use pattern** ^a^	
After cleaning	144(35.64)
Without cleaning	37(9.16)
Single use	223(55.20)
**Multiple mask** ^b^	
No	43(27.22)
Yes	115(72.78)
**History of COVID-19** ^b^	
No	94(59.49)
Yes	64(40.51)
**COVID-19 Vaccination** ^b^	
Not Vaccinated	20(12.66)
1^st^ Dose	11(6.96)
Both Dose	127(80.38)
**Workplace AC** ^b^	
No	97(61.39)
Yes	61(38.61)

^a^Participants responding to these questions (N) = 404.

^b^Participants responding to these questions (N) = 158.

All data presented as N(%).

### Dermatological problems

**Fig 2:**
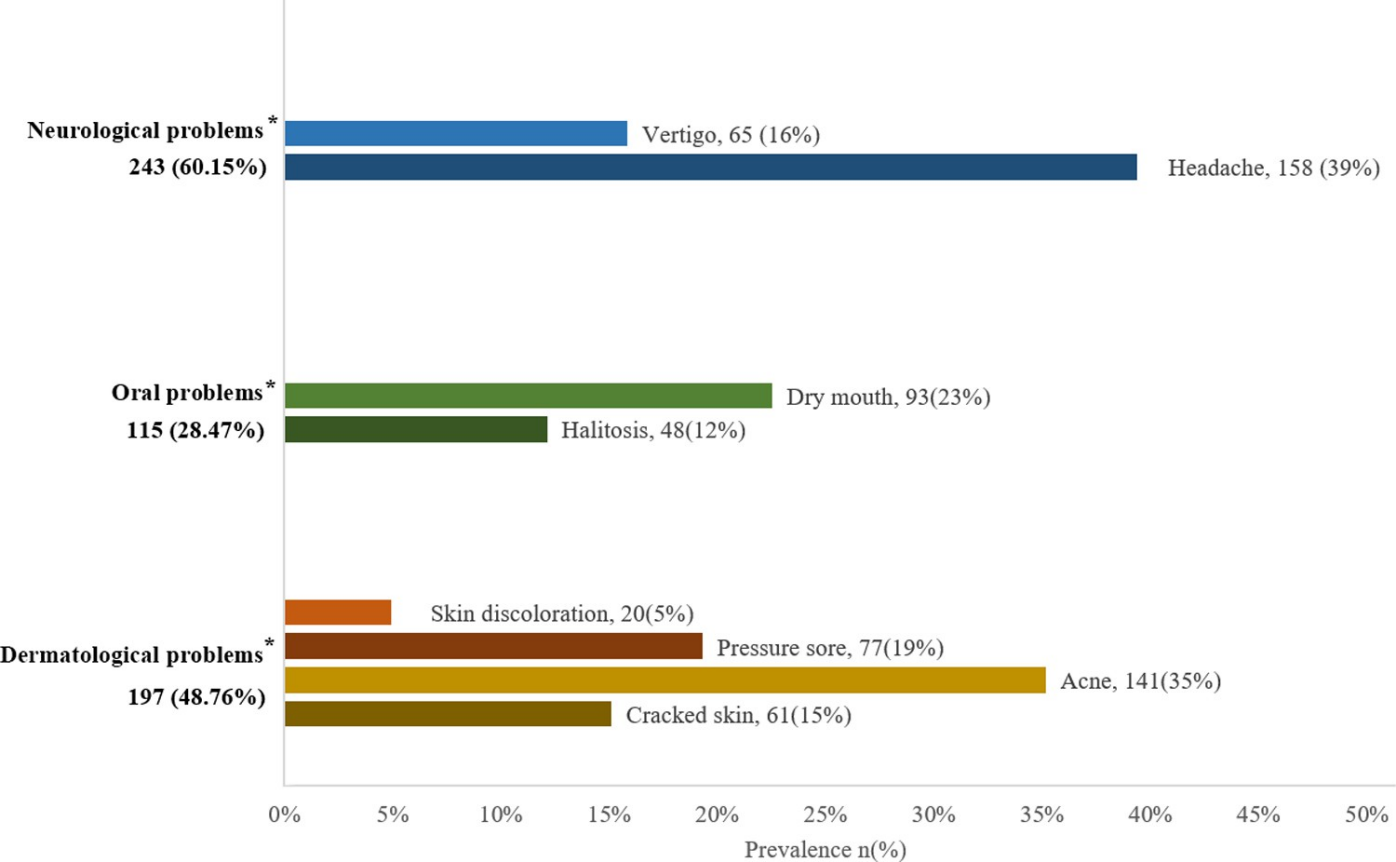
Types of dermatological, oral & neurological problems following face mask use (n = 404). *Multiple Responses.

There were unfavorable skin responses on the skin beneath the face masks in 197(48.76%) cases. Acne, skin discoloration, cracked skin, pressure sore were dermatological problems reported by the participant (Fig 2).

We performed a bivariate analysis, and the unadjusted result is provided in Table 2. The chi square test shows that gender was significantly aassociated to dermatological problems, with females being more prone to these issues (63.93%). Workplace was also significantly associated with dermatological problems, with individuals working in government institutions (59.06%) being more susceptible to these diseases. The average weekly mask usage was significantly associated with dermatological disorders, and those who worked more than 56 hours per week (63.27%) were more likely to have these problems. The N95 surgical mask and cloth mask were significantly associated with dermatological disorders among facemask types, with 52.70% of N95 mask users, 51.01% of surgical mask users, and 70.91% of cloth mask users more likely to have these problems. Multiple mask usage was also significantly connected with dermatological difficulties, with 67.83% of multiple mask users having these issues. COVID-19 history was significantly related with dermotilogical difficulties, and individuals with a positive COVID-19 history were more prone to this problem (Table 2).

**Table 2:** Prevalence of dermatological, oral and neurological problems from face mask use.

Variable	N (%)	Dermatological problem (Acne, Skin discoloration, Skin crack, Pressure sore)			Oral problem (Dry mouth, halitosis)			Neurological problem (Vertigo, Headache)		
		No 207(51.24)	Yes 197(48.76)	P-value	No 289(71.53)	Yes 115(28.47)	P-value	No 161(39.85)	Yes 243(60.15)	P-value
**Age** ^a^										
<26 years	80(19.80)	37(46.25)	43(53.75)	0.483	59(73,75)	21(26.25)	0.740	32(40.00)	48(60.00)	0.933
26–36 years	284(70.30)	147(51.76)	137(48.24)		200(70.42)	84(29.58)		112(39.44)	172(60.56)	
> 36 years	40(9.90)	23(57.50)	17(42.50)		30(75.00)	10(25.00)		17(42.50)	23(57.50)	
**Occupation** ^a^										
Doctor	301(74.50)	153(50.83)	148(49.17)	0.074	213(70.76)	88(29.24)	0.455	118(39.20)	183(60.80)	0.570
Nurse	51(12.62)	21(41.18)	30(58.82)		35(68.63)	16(31.37)		19(37.25)	32(62.75)	
Technologist	31(7.67)	21(67.74)	10(32.26)		26(83.87)	5(16.13)		16(51.61)	15(48.39)	
Others	21(5.20)	12(57.14)	9(42.86)		15(71.43)	6(28.57)		8(38.10)	13(61.90)	
**Sex** ^a^										
Female	122(30.20)	44(36.07)	78(63.93)	**<0.001**	84(68.85)	38(31.15)	0.432	36(29.51)	86(70.49)	**0.005**
Male	282(69.80)	163(57.80)	119(42.20)		205(72.70)	77(27.30)		125(44.33)	157(55.67)	
**Workplace** ^a^										
Unemployed	35(8.66)	26(74.29)	9(25.71)	**<0.001**	27(77.14)	8(22.86)	0.836	23(65.71)	12(34.29)	**0.001**
Government facilities	171(42.33)	70(40.94)	101(59.06)		125(73.10)	46(26.90)		52(30.41)	119(69.59)	
Private facilities	104(25.74)	56(53.85)	48(46.15)		72(69.23)	32(30.77)		43(41.35)	61(58.65)	
Dental chamber	32(7.92)	25(78.13)	7(21.88)		24(75.00)	8(25.00)		19(59.38)	13(40.63)	
ICU	3(0.74)	2(66.67)	1(33.33)		2(66.67)	1(33.33)		1(33.33)	2(66.67)	
Others	59(14.60)	28(47.46)	31(52.54)		39(66.10)	20(33.90)		23(38.98)	36(61.02)	
**Duration of mask use** ^a^										
≤12 months	89(22.03)	52(58.43)	37(41.57)	0.124	70(78.65)	19(21.35)	0.092	40(44.94)	49(55.06)	0.266
>12 months	315(77.97)	155(49.21)	160(50.79)		219(69.52)	96(30.48)		121(38.41)	194(61.59)	
**Average mask use/week** ^a^										
< 32 hours	163(40.35)	109(66.87)	54(33.13)	**<0.001**	120(73.62)	43(26.38)	0.600	82(50.31)	81(49.69)	**0.001**
32–56 hours	143(35.40)	62(43.36)	81(56.64)		98(68.53)	45(31.47)		44(30.77)	99(69.23)	
> 56 hours	98(24.26)	36(36.73)	62(63.27)		71(72.45)	27(27.55)		35(35.71)	63(64.29)	
**Facemask type**										
**N95** ^a^										
No	208(51.49)	118(56.73)	90(43.27)	**0.023**	159(76.44)	49(23.56)	**0.024**	92(44.23)	116(55.77)	0.064
Yes	196(48.51)	89(45.41)	107(54.59)		130(66.33)	66(33.67)		69(35.20)	127(64.80)	
**KN95** ^a^										
No	256(63.37)	137(53.52)	119(46.48)	0.228	182(71.09)	74(28.91)	0.796	102(39.84)	154(60.16)	0.997
Yes	148(36.63)	70(47.30)	78(52.70)		107(72.30)	41(27.70)		59(39.86)	89(60.14)	
**Surgical** ^a^										
No	57(14.11)	37(64.91)	20(35.09)	**0.026**	41(71.93)	16(28.07)	0.943	25(43.86)	32(56.14)	0.505
Yes	347(85.89)	170(48.99)	177(51.01)		248(71.47)	99(28.53)		136(39.19)	211(60.81)	
**Cloth mask** ^a^										
No	349(86.39)	191(54.73)	158(45.27)	**0.000**	255(73.07)	94(26.93)	0.086	144(41.26)	205(58.74)	0.145
Yes	55(13.61)	16(29.09)	39(70.91)		34(61.82)	21(38.18)		17(30.91)	38(69.09)	
**Other mask** ^a^										
No	379(93.81)	196(51.72)	183(48.28)	0.455	272(71.77)	107(28.23)	0.686	152(40.11)	227(59.89)	0.685
Yes	25(6.19)	11(44.00)	14(56.00)		17(68.00)	8(32.00)		9(36.00)	16(64.00)	
**Use pattern** ^a^										
After cleaning	144(35.64)	65(45.14)	79(54.86)	0.189	97(67.36)	47(32.64)	0.335	49(34.03)	95(65.97)	0.155
Without cleaning	37(9.16)	20(54.05)	17(45.95)		26(70.27)	11(29.73)		18(48.65)	19(51.35)	
Single use	223(55.20)	122(54.71)	101(45.29)		166(74.44)	57(25.56)		94(42.15)	129(57.85)	
**Multiple mask** ^b^										
No	43(27.22)	25(58.14)	18(41.86)	**0.003**	29(67.44)	14(32.56)	0.356	19(44.19)	24(55.81)	0.160
Yes	115(72.78)	37(32.17)	78(67.83)		86(74.78)	29(25.22)		37(32.17)	78(67.83)	
**History of COVID-19** ^b^										
No	94(59.49)	45(47.87)	49(52.13)	**0.007**	74(78.72)	20(21.38)	**0.042**	45(47.87)	49(52.13)	**<0.001**
Yes	64(40.51)	17(26.56)	47(73.44)		41(64.06)	23(35.94)		11(17.19)	53(82.81)	
**COVID-19 Vaccination** ^b^										
Not Vaccinated	20(12.66)	9(45.00)	11(55.00)	0.753	18(90.00)	2(10.00)	**0.013**	7(35.00)	13(65.00)	0.771
1^st^ Dose	11(6.96)	5(45.45)	6(54.55)		11(100)	0(0)		5(45.45)	6(54.55)	
Both Dose	127(80.38)	48(37.80)	79(62.20)		86(67.72)	41(32.48)		44(34.65)	83(65.35)	
**Workplace AC** ^b^										
No	97(61.39)	38(39.18)	59(60.82)	0.983	76(78.35)	21(21.65)	**0.047**	33(34.02)	64(65.98)	0.637
Yes	61(38.61)	24(39.34)	37(60.66)		39(63.93)	22(36.07)		23(37.70)	38(62.30)	

^a^Participants responding to these questions (N) = 404.

^b^Participants responding to these questions (N) = 158.

All data presented as N(%).

p-values <0.05 are in bold.

Risk factors were further examined for multivariate logistic regression (Table 3). The result revealed that females were 3.4 times more likely to suffer from dermatological problems due to mask use than males (Adjusted Odd Ratio [AOR] = 3.41; 95% CI: 0.113–0.765; p = .012). Doctors were most likely to suffer from skin reactions, and compared to them, nurses and technologists had 78% (AOR = 0.217, 95%CI: .067-.705) and 48% (AOR = 0.515; 95% CI: 0.11–2.38; p = .011) less risk, respectively. Wearing a face mask for 32–56 hours/week and >56 hours/week increased the risk of skin reactions by three (AOR = 2.9, 95%CI: .981–8.577) and four times (AOR = 4.06; 95% CI: 1.36–12.12; p = .012) respectively compared to wearing a mask for <32 hours. Participants who used N95 masks were 2.5 times (AOR = 2.54; 95% CI: 1.063–6.073; p = .036), and those who used multiples masks were 2.9 times (AOR = 2.9; 95% CI: 1.184–7.103; p = .02) at risk of having dermatological problems. Furthermore, individuals with a previous history of COVID-19 infection were 3.6 times more likely to have dermatological problems (AOR = 3.6; 95% CI: 1.529–8.586; p = .003).

**Table 3:** Factors associated with dermatological, oral, and neurological problems.

Variables	Dermatological problem (Acne, Skin discoloration, Skin crack, pressure sore)				Oral problem (Dry mouth, halitosis)				Neurological problem (Vertigo, Headache)			
	AOR	p- value	95% CI		AOR	p- value	95% CI		AOR	p-value	95% CI	
**Age**												
<26 years	1				1				1			
26–36 years	.716	.532	.251	2.043	.955	.933	.331	2.761	2.602	.084	.88	7.693
> 36 years	1.139	.855	.281	4.619	.677	.617	.147	3.128	4.326	.055	.969	19.312
**Occupation**												
Doctor	1				1				1			
Nurse	.217	**.011**	.067	.705	.454	.191	.139	1.485	.112	**.001**	.03	.418
Technologist	.515	.396	.111	2.386	.992	.992	.202	4.875	.18	**.033**	.037	.874
Others	.746	.71	.159	3.492	.971	.973	.173	5.461	.367	.251	.067	2.029
**Sex**												
Male	1				1				1			
Female	3.407	**.012**	1.307	8.880	4.073	**.009**	1.430	11.597	6.913	**<0.001**	2.436	19.614
**N95 mask use**												
No	1				1				1			
Yes	2.541	**.036**	1.063	6.073	3.708	**.005**	1.498	9.181	1.328	.521	.559	3.154
**Mask use pattern**												
After cleaning	1				1				1			
Without cleaning	.227	.086	.042	1.231	.055	**.029**	.004	.747	.348	.24	.06	2.023
Single use	.492	.115	.203	1.189	.292	**.008**	.118	.725	.755	.53	.313	1.816
**Multiple mask use**												
No	1				1				1			
Yes	2.9	**.02**	1.184	7.103	.281	**.017**	.099	.798	1.264	.613	.51	3.138
**Weekly mask use**												
< 32 hours	1				1				1			
32–56 hours	2.901	.054	.981	8.577	.993	.991	.29	3.402	.736	.588	.242	2.236
> 56 hours	4.06	**.012**	1.36	12.122	1.104	.874	.326	3.743	.796	.683	.266	2.383
**History of COVID**												
No	1				1				1			
Yes	3.624	**.003**	1.529	8.586	2.691	**.024**	1.14	6.351	5.795	**<0.001**	2.348	14.306

AOR = Adjusted Odds Ratio, CI = Confidence Interval.

p-values <0.05 are in bold.

### Oral problems

The prevalence of oral problems among face mask users was 28.47%. Halitosis and dry mouth were the reported oral problem by the participants (Fig 2). In bivariate analysis of facemask types, the N95 surgical mask was significantly associated with oral problems, with 33.67% of N95 mask users having these problems. COVID-19 history was significantly associated with oral problems, and participants (35.94%) with a positive history of COVID-19 were more prone to this problem. COVID-19 vaccination was also linked to oral problems, and 32.48% who had two doses of the vaccine said they had these problems (Table 2).

Multivariate logistic regression (Table 3) revealed that females were four times more likely to suffer from oral problems than males (AOR = 0.293; 95% CI: 0.086–0.699; p = .009). Those who used face masks one time only (AOR = 0.292; 95% CI: 0.118–0.725; p = .008) and multiple masks simultaneously (AOR = 0.281; 95% CI: 0.099–0.798; p = .017) had a lower chance of having oral problems. Oral problems were less likely to occur in individuals residing increasing duration in humid areas (AOR = 0.029; 95% CI: 0.002–0.453; p = .012).

### Neurological problems

60.15% of all the study participants suffered from neurological problems. The neurological problems included headaches and vertigo (Fig 2). In bivariate analysis, gender was significantly associated with neurological problems, with females being more prone to these issues (70.49%). The workplace was also associated to neurological issues, with those working in government organizations (69.59%) being more vulnerable. The average weekly mask wear was linked to neurological disorders, and those who worked 32 to 56 hours per week (69.23 percent) were more likely to have these issues. COVID-19 history was linked to neurological issues, and those with a positive COVID-19 history (82.81 percent) were more likely to have this condition (Table 2).

After fitting the multivariate regression model (Table 3), we found out females were 6.9 times more likely to suffer from neurological problems. We also found out doctors were the most likely victims of neurological problems, compared to the nurses, technologists and other staffs were 89% (AOR = 0.112; 95% CI: 0.03–0.418), 82% (AOR = 0.18; 95% CI: 0.037–0.874) and 63% (AOR = 0.367; 95% CI: 0.067–2.029; p = .001) less at risk, respectively. Individuals who had previous COVID-19 infection reported 5.8 times more neurological problems than those without (AOR = 5.795; 95% CI: 2.348–14.306; p = < .001).

## Discussion

The COVID-19 outbreak has had a devastating effect on the physical and emotional well-being of both the general populace and emergency personnel worldwide. Health care workers (HCW) have been putting their lives on the line to safeguard patients and their families since the outbreak began [17]. To prevent the spread of the virus during this pandemic, World Health Organization (WHO) is asking to wear hygienic masks for both HCWs and the general population. The Health Department of the Bangladesh government has made it mandatory to wear a mask when outside on May 8, 2020. As time progressed, many problems caused by prolonged mask use have been noticed, especially among HCWs.

A study conducted in China in March 2020 found that 98.03% of first-line health workers had dermatological problems due to PPE. The nasal bridge was affected in 63% of cases on that [18]. A similar study in Thailand found that in 54.25% of cases, the mask caused adverse skin reactions, which included both HCWs and the general population [19]. In our study, the prevalence of adverse skin manifestations (acne, skin discoloration, skin crack, pressure sore) was 51.24%. The warm-muggy air in the dead space under the mask, in comparison to the ambient temperature, was the principal cause of such problems. Our study also revealed that females were 3.4 times more likely to suffer from mask-induced dermatological problems. More than half of females in our study reported dermatological problems similar to another study (53.4%) done in Pakistan [9]. Besides, wearing a face mask can accentuate dermatological problems (i.e., autoimmune diseases, allergic diseases, pigment disorders, and hair diseases), which are generally more common in females [20]. It can be explained by a range of aspects, including hormonal factors, pH, skin thickness, sweat rate, and other aspects, which vary widely between males and females [21, 22].

Lan J at el. proposed that the duration of mask use more than 6 hours/day was a risk factor of developing mask-related dermatological problems [18]. Another study also found that wearing a mask for more than 4 to 8 hours per day and more than 8 hours showed a higher risk of adverse skin reactions [23]. Our study also found that wearing a face mask for 32 to 56 hours/week and more than 56 hours/week increased the risk of skin reactions by three and four times, respectively. In prior research, excessive mouth perspiration was found in 67.6% of healthcare professionals who wear masks for a long time. HCW are more likely to touch the facemasks because of the irritation they produce, which may lead to contamination of the hands [24]. Kaihui et al. [12] reported 95.1% adverse skin reactions such as nasal bridge scarring, dry skin, and rash among N95 mask users, which 54.59% in this study. Our research found that N95 mask users have a 2.5 times higher risk of dermatological problems, whereas, for KN95 mask users, we could not find any such amplified impact. In previous research, it was reported that N95 respirators were 8-fold thicker and had a 2-fold higher dipole charge density than that of KN95 respirators [25]. So, it’s natural that the outward flow from the face through N95 is less possible, which can cause the loss of integrity of skin tissues. On the other hand, surgical mask-induced adverse skin reaction was 51.01% which was quite similar to a study in Thailand (67.62%), although that study was conducted on the general population [19]. The CDC recommended using a cloth mask with multiple layers of fabric or wearing a disposable mask underneath a cloth mask to improve mask efficiency [26]. Hence there was a trend of using multiple masks simultaneously, which was prevalent in 73% of our participants who showed a 2.9 times higher chance of developing adverse skin reactions. Multiple mask user (p = 0.003) had a great association with dermatological problem which was found in our study Although the mask type used in this trend masks was not recorded. Techasatian et al. found a 1.5 times greater chance of developing adverse skin manifestations in the case of re-using the mask at least for 2–3 days, although our study found none [19]. Individuals with a previous history of COVID-19 infection were 3.6 times more likely to have dermatological problems which support other studies [19, 27, 28]. There is still inadequate research on this issue. Prior research has clear indication that, the prolonged use of face mask triggers primary facial tissue damage which finally induces loss of facial skin integrity. This loss ultimately creates entry-point for pathogens including the coronavirus etc. [29]. However, petroleum jelly, a widely accepted and reasonably priced skin protectant, has been shown in biotribological studies to lower Skin’s the coefficient of friction (COF) by around 25% immediately after application, the COF value eventually rises to its pre-application level again after about an hour [29].

Due to continuous use of the face mask, 35.3% participants in an Indian research had dry mouth and 22.4% acquired halitosis [24]. These problems are noted in 28.47% of our study population. Duration of face mask-wearing, N95 mask use, history of COVID-19 infection, COVID-19 vaccination, and availability of workplace cooling system was associated with oral problems (dry mouth, halitosis). Because of continuous use of the facemask, 35.3% of participants had dry mouth and 22.44% developed halitosis [24]. In our study, we also found that 30.48% people who use masks for more than 12 months face dry mouth and halitosis. Also, people who re-use masks without cleaning and never re-use at all tended to have fewer oral problems than those who re-use masks after cleaning. The cleaning agents and technique can be a factor in this issue which warrants further investigation. There was a previous research that revealed N95 respirators could be decontaminated and reused, but that integrity of respirator fit and seal had to be maintained [30]. Also, an air-cooling system in the workplace can reduce the oral problems associated with face mask wearing by controlling the humidity of the environment. A humid environment causes increased sweating and dehydration, which eventually reduces saliva water and can finally cause dry mouth and halitosis. Oral problems are nearly four times more common among the N95 mask user. N95 may form a tight barrier that prevents normal nasal breathing, forcing a person to breathe through their mouth. Mouth breathing may disturb oral flora, resulting in oral problems, increased caries risk, and halitosis [31].

60.15% of all study participants suffer from neurological problems included headaches and vertigo, because of face mask use. We found out females were 6.9 times more likely to suffer from neurological problems like headache and vertigo as headache-related disabilities are generally common in females [32, 33]. According to this study, neurological issues were reported 5.8 times more common among the COVID-19 sufferers. This can be interpreted as a post-COVID-19 complication but still needs further investigation. Severe headaches have been associated with extensive external compression of pericranial soft tissues by tight bands or straps around the head (e.g. face mask). Face masks have been associated to de novo headaches in several studies [34]. Our study showed 60.64% of the respondent were victim of face mask induced headache. Frequent short breaks, neck massage, and keeping hydrated, especially before starting duty, can help alleviate this problem [35].

It’s essential to note some of our study’s limitations as well as the methods we used to solve them. Due to our study’s cross-sectional nature, we cannot infer causality for the associations that we have presented in this paper. However, by presenting AORs using multiple regression models, we attempted to account for the potential effect of confounders. All the problems presented in this study were self-reported by health care workers, which may lead to self-reporting bias. In order to minimize this, all our data collectors were medical students and they were trained by experts before data. Besides, several types of materials might have been used to manufacture the masks included in the study and therefore we could establish association to the mask type only, not to the material used. We also acknowledge that the findings of our study may not be generalizable due to our sample size, but we selected the healthcare facilities randomly to make the sample representative and reduce selection bias.

## Conclusion

In conclusion, the use of face masks contributes significantly to the discomfort experienced by all participants after continuous use, which might restrict the mask’s effectiveness, resulting in diminished protection. Nevertheless, use of face mask is completely unavoidable to control the spread of COVID-19. Prolonged exposure to N95 and wearing more than one mask were associated with skin issues. So, to avoid the negative impacts of prolonged use of face masks we need to follow the best practices of face mask usage such as not using single use mask multiple times, properly cleaning multi usage mask before each reuse, using skin comforting agent to reduce skin irritation etc.

## Supporting information

S1 Dataset Dataset used to generate figures, graphs, tables, and statistics. (DTA)
